# Analysis of T-Cell Receptor Repertoire in Transplantation: Fingerprint of T Cell-mediated Alloresponse

**DOI:** 10.3389/fimmu.2021.778559

**Published:** 2022-01-12

**Authors:** Guangyao Tian, Mingqian Li, Guoyue Lv

**Affiliations:** Department of Hepatobiliary and Pancreatic Surgery, The First Hospital of Jilin University, Changchun, China

**Keywords:** alloreactive, T-cell receptor repertoire, transplant, biomarker, high-throughput sequencing

## Abstract

T cells play a key role in determining allograft function by mediating allogeneic immune responses to cause rejection, and recent work pointed their role in mediating tolerance in transplantation. The unique T-cell receptor (TCR) expressed on the surface of each T cell determines the antigen specificity of the cell and can be the specific fingerprint for identifying and monitoring. Next-generation sequencing (NGS) techniques provide powerful tools for deep and high-throughput TCR profiling, and facilitate to depict the entire T cell repertoire profile and trace antigen-specific T cells in circulation and local tissues. Tailing T cell transcriptomes and TCR sequences at the single cell level provides a full landscape of alloreactive T-cell clones development and biofunction in alloresponse. Here, we review the recent advances in TCR sequencing techniques and computational tools, as well as the recent discovery in overall TCR profile and antigen-specific T cells tracking in transplantation. We further discuss the challenges and potential of using TCR sequencing-based assays to profile alloreactive TCR repertoire as the fingerprint for immune monitoring and prediction of rejection and tolerance.

## Introduction

T cells recognize antigens through their TCRs binding to antigen-presenting cells (APCs) surface peptide-major histocompatibility complex (pMHC) (1). The binding will result in changes in the CD3 molecule complex and initiate downstream signaling pathways, which are called TCR triggering. Subsequently, cells with foreign or self-mutated pMHC will be distinguished and eliminated by T cells (2, 3).

The T-cell repertoire covering the enormous foreign and self-mutated antigens is achieved *via* highly polymorphic TCRs (4, 5). As the determining factor of antigen-specificity and the potential of being used as the molecular identifiers of T cells, TCRs receive special attention in many fields of diseases, such as infectious diseases, autoimmune diseases, and malignant tumors (5–7). In immune processes, TCR repertoire analysis provides information on the T cells dynamics with respect to diversity and clonality. The characterization of TCR repertoires in transplantation can depict the T-cell dynamics at multiple timepoints, and recipient or donor antigen-specific TCR repertoires can be used for fingerprint recognition in tracing the alloresponse and assisting with diagnosis and treatment.

## T Cells in Transplantation

During the T cell selection process in the thymus, the affinity between TCRs and self-pMHC molecules play a central role. Only those T cells with appropriate binding affinity with self-MHC and do not recognize self-antigen will survive in the positive and negative selection. This is the basis of T cell distinguishing foreign antigens from self-antigens (8, 9).

Following the upregulation of S1P1, T_N_(naïve T cell) cells enter the periphery *via* lymphatics or blood vessels (10), and express a series of typical molecules, including CD62L and CCR7, which are necessary for the subsequent trafficking of T_N_ cells between the secondary lymphoid organs (11, 12). After being exposed to antigens, T_N_ cells have no immediate effector functions. The activation and proliferation of T_N_ cells depend on signals. The first signal is received through TCR engagement with pMHC complexes and the second signal is relayed through costimulatory receptors, of which CD28 is dominant. After the signals cross the activation threshold, T_N_ cells are activated, and will initiate clonal expansion and effector differentiation to acquire capacity to eliminate target cells by recognizing the alloantigen. A small portion of the T cells can differentiate into long-lived memory cells. When encountering the same antigen again, memory T cells can be activated with limited co-stimulation signal and respond to lower doses of antigen compared with T_N_ cells (13, 14). Actually, the memory T-cell repertoires in adult human contain high frequencies of pre-existent alloreactive memory T cells that are able to infiltrate the graft rapidly and cause rejection through alloresponse (14–16). These alloreactive memory T cells, mainly CD8 T cells (17), can locate within the lymph nodes (central memory T cell, T_CM_), peripheral non-lymphoid tissues (resident memory T cell, T_RM_), or periphery (effector memory T cell, T_EM_) and stay resting until they encounter the same antigen again post-transplantation. Through quantitative analysis of alloresponse *in vitro*, up to 10% of peripheral T cells were considered potentially alloreactive (18, 19).

There are many mechanisms for the generation of pre-existing alloreactive memory T cells before transplantation. Humans exposed to alloantigens through previous transplantation, blood transfusion, or pregnancy may produce alloantigen-specific memory T cells (20–22). Without contact with allogeneic MHC molecules, the exposure to commensal bacteria or environmental antigens is also probably to induce potent heterologous immunity, which is one way for the generation of alloreactive memory T cells (23, 24). Many studies have identified that alloreactive T-cell clones can also stem from other immune events (25, 26), such as dozens of virus-specific (CMV, EBV, Flu, HIV, Zika Virus, SARS-CoV-2) memory T cells that show cross-reactivity to allogeneic pMHC complexes (27–32). Thereby, these memory T cells can also pre-exist in the host even if they have not previously encountered donor-derived antigens.

A series of studies have indicated that both naïve and memory alloreactive T cells are the mediators of the alloreactive immune response in transplantation (33, 34). They make contribution to both acute and chronic rejection episodes depending on the pathway recognizing donor antigens (35, 36). Host T cells recognizing processed donor allogeneic peptides presented by host MHC is indirect allorecognition (37). Allogeneic T cells that recognize nonself MHC molecules from the same species are also responsible for the alloresponse in allotransplantation, called direct T-cell allorecognition (38). In some settings, intact donor MHC can be transferred to the surface of recipient APCs, activation of recipient allogeneic T cell by the engagement of recipient TCRs with this donor-derived MHC on recipient APC is called semidirect pathway (39, 40). Direct pathway responses are considered strong but only last a short time and likely mediate the acute rejection, whereas indirect pathway responses are viewed as be more long-term and responsible for mediating chronic rejection. These pathways may be involved in mediating allograft rejection at the same time or at different times. During the process of transplantation, both alloreactive naïve T cells and pre-existing memory T cells are exposed to high and long-lasting alloantigen loads to mediate rejection through direct and indirect pathways. Indirect pathway-mediated rejection largely depends on the T_N_ repertoire, while direct pathway-mediated rejection involves both naïve and memory T cells (41). Oberbarnscheidt et al. reported that T_EM_ played an important role in immune surveillance of grafts and had no need to return to secondary lymphoid tissues to differentiate for effector functions in mouse model (42). In humans, through measuring peripheral T cells from healthy donors based on flow-cytometry, Macedo and colleagues reported the proliferation rate of CD4^+^ and CD8^+^ T_N_ cells under the stimulation from allogeneic cells was similar with their memory counterparts *in vitro (* 43). Similar findings were also reported in mouse model for direct allorecognition by naïve and memory T cells (44, 45).

Although the contribution is small, alloreactive T cells can also derive from dual TCR T cells with two distinct TCR α chains that give one T cell two distinct antigen-specificities and double the chance of antigen recognition (46, 47). In studies of mice, it has been shown that dual TCR T cells have a high frequency of alloresponse (48). In acute graft versus-host disease (GVHD) patients, the frequency of dual receptor T cells is 5.3 times higher than that in healthy controls (49).

To suppress rejection or to induce tolerance, treatment is usually targeted at both naïve and memory T cells. Long-term administration of immunosuppressive drugs after transplantation is the most common therapeutic option for depleting naïve and memory T cells or preventing their full activation for acute cellular rejection (ACR). A similar effect can be induced by alloreactive T-cell exhaustion. Long-term or large loads of foreign antigen exposure to T cells may lead to antigen-specific T-cell exhaustion (50). During the first 6 to 12 months post-transplantation, the faltering demand for immunosuppressive therapy as well as the decline in the incidence of ACR coincided with the increase in the frequency of exhausted T cells (T_EXH_) (51). Fribourtg et al. demonstrated that T_EXH_ development was correlated with better clinical outcomes (52). Another way to induce tolerance is through the adoptive transfer of regulatory T cells (Tregs). Tregs are a suppressive subset of T cells and can inhibit the function of conventional T cells and other immune cells (53), and have been well demonstrated to induce tolerance in various types of transplantations (54–57). For example, in solid organ transplantation clinical trials, polyclonal Tregs had been proved to be safe and effective in alloresponse repression. Antigen-specific Treg therapies, such as Treg engineered with antigen-specific TCRs (TCR-Tregs) and chimeric antigen receptor (CAR)-modified Tregs (CAR-Tregs), further overcame the shortage of a limited number of ex vivo Tregs expansions by performing the suppressive function in a more effective and antigen-specific way on alloreactive T cells (58–60).

## TCR in Alloresponse

**Figure 1:**
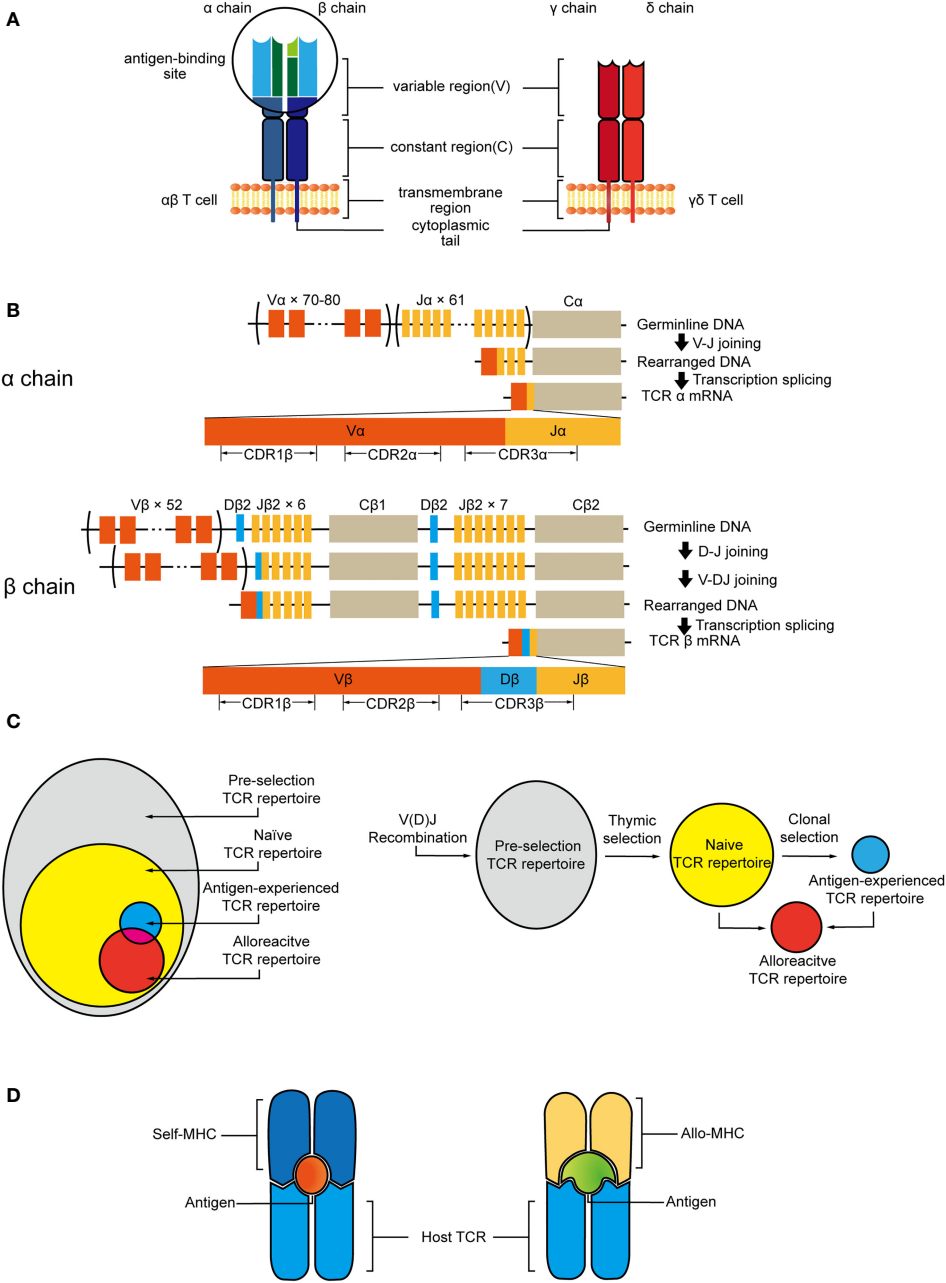
**(A)** αβ TCR consists of α and β chain, γδ TCR is composed of γ and δ chain. Each subunit has constant regions and variable regions that are site of antigen recognition. **(B)** V, D, and J gene segments are progressively rearranged to form the final DNA sequence. This variability is further increased by deletion and insertion of nucleotides at the junction sites. After transcription, the sequence between the recombined V(D)J regions and the gene encoding the C region will be spliced out. V gene encodes CDR1 and CDR2, whereas junction of the V(D)J genes encodes CDR3. **(C)** Pre-selection TCR repertoire is stochastic and diverse. Thymic selection purges most of clones and shapes the naive TCR repertoire which contains alloreactive clones. Antigen exposure leads to clonal expansion of antigen-specific T-cell clones that may also have the potential of the cross-recognition with allo-antigen/MHC. **(D)** Allo-MHC could be recognized by host-TCR through imitating the unique features of host-pMHC complex.

The specificity of T cells is determined by their TCRs (4, 61). The TCR consists of two distinct chains. For human beings, the overwhelming majority (95%) of circulating T cells express TCRs composed of an α chain and β chain. And there is a small subset, the TCR of which is composed of a γ chain and δ chain (62, 63) (**Figure 1A**). αβ T cells are the central mediators of the adaptive immunity with great diversity, recognizing antigens presented related to MHC Class I and II proteins. To cover a wide range of potential pathogens and harmful substances, billions of unique TCR molecules need to be generated *in vivo*, and this extraordinary heterogeneity of TCRs is achieved through their variable region. Both chains of TCRs include a highly variable complementarity determining region 3 (CDR3), which are the binding sites with MHC peptide and mediate TCR engagement (64). The rearrangement of variable (V), joining (J), and diversity (D) gene segments and the random insertion or deletion of nucleotides at the junction of gene segments leads to generation of variable exons, that is the major factor contributing to the diversity of TCR sequences (65, 66) (**Figure 1B**). The theoretically estimated number of possible gene combinations generated during the lifespan of human beings is 10^15^ to 10^20^ (67).

V(D)J recombination influenced by both genetic and epigenetic factors leads to the formation of pre-selection TCR repertoire (68, 69). During the thymus selection, the TCR repertoire will be selected by self-MHC ligand and antigen. Thymic selection will purge the majority of clonotypes and only a few T cells that are granted access to the periphery to make up the naïve TCR repertoire. T cells selected by self-MHC, and clones bearing TCRs within additional affinity to allo-MHC will not be removed during this process. Thus, the process initially shapes a naïve T cell repertoire containing T cells that have the potential of interacting with allo-antigen/MHC (**Figure 1C**).

Moreover, recent experimental evidence also supports that each T cell has the potential to interact productively with more than one pMHC complex (70). The cross-reactivity of TCRs on specificity not only provides potential immune coverage but also increases the risk for transplant rejection. The clones from antigen-experienced TCR repertoire could also be involved in alloresponse (**Figure 1C**). Distinct pMHC complexes could have a similar surface conformation due to the shared conformation between MHC molecules and peptides. Therefore, allogeneic pMHC could be recognized by TCRs through imitating the unique features of peptides presented by self-MHC (71). The overlap between alloreactive clones and known public virus-reactive clones has been proved by the shared TCR sequences of them. And crystal structures have shown that due to the plasticity of the CDR loops, TCR could adapt to structurally diverse pMHC ligand by small conformational changes or rearrangements of its central CDR loops, this recognition can be driven by unique features of both the peptides and allo-MHC molecules (64, 72, 73) (**Figure 1D**). Recent *in vivo* finding dictates that the self-peptides bound to a handful of allogeneic MHC-peptide complexes account for a large proportion of the alloresponse, and the complex of self-peptide and allo-MHC molecule plays a critical role in transplant tolerance induction (74).

Many examples of TCR bias have been observed in various diseases. Based on the CDR3 sequences, the T cell clones within different tissues, timepoints, individuals, and even species are able to be traced and quantified (75, 76).The analysis of total CDR3 lengths and distribution has been adopted to measure the degree of clonality and diversity of T cells during the immune response. Certain clonal expansion and reduction also exhibit T cell-mediated immune responses caused by specific antigens (77). In addition, these sequences can be identified by matching with a reference antigen-specific TCR repertoire (78). The unique TCR repertoires are used as the fingerprints to monitor immune status and predict response.

Through one-way mixed lymphocyte reaction (MLR) using responder and stimulator cells from different individuals, Emerson et al. first characterized the alloreactive TCR repertoire in healthy adults (79). However, there is no unique CDR3 length or gene usage in alloreactive T cell populations compared with other non-alloreactive clones, which means that there may not be a unique feature of the alloreactive TCR repertoire. Although there was a small amount of highly abundant clones in the alloreactive clones, likely memory clones, the frequencies of most alloreactive clones in circulation were at naïve levels, which further supports the high diversity of alloreactive TCR repertoire. Indeed, the T_N_ repertoire does have a far higher diversity compared to the memory counterparts (80, 81), as the repertoires of memory T cells have contracted due to the previous clonal expansion, while the naïve pool has not been exposed to antigens and are less affected. Therefore, it is understandable that the repertoire of unprimed T_N_ cells has better coverage of allogeneic pMHC. Moreover, another *in vitro* experiment showed that the alloreactive TCR repertoires in the same recipient were disparate when the donor antigens were from two individuals (19, 82).

Unlike some pathogen antigen-specific TCR repertoire which has a preferential usage of V and J gene segments and dominant CDR3 length (83), the lack of uniform feature and the diversity of the alloreactive CDR3 hinder the characterization of alloreactive TCR repertoire. Fortunately, TCR sequencing combined with *in vitro* MLR that proved to be reliable and sensitive opens up a new way for annotating alloreactive TCR sequences and capturing alloresponse (79).

## High-Throughput Technology Revolutionized Methods for Measuring Alloresponse

To characterize antigen-specific T cells in human tumors, infectious diseases, and transplantation, various low-throughput methods have been proposed and developed (84). However, these low-throughput methods screen a limited number of T or B cells against a few antigens at one time (85).

Monoclonal antibodies against the Vβ chain of TCRs to describe the T-cell repertoires at the protein level, which enabled qualitative and quantitative determination. However, limited to the types of antibodies with specific recognition site, this method offers less coverage and it does not reveal any information on CDR3 diversity (86, 87). The distribution of CDR3 sequence length in the entire T-cell repertoire was first exhibited through CDR3 spectratyping (88). And Sanger sequencing was applied to measure the TCR repertoire at the gene level and describe it in more detail (89, 90). But it was not enough to determine the diversity of the entire TCR repertoire, because only a few high sequences could be captured to construct the library (91).

NGS technology has made significant progress in the field of TCR analysis (92, 93), which introduced high-throughput, and ultra-sensitive techniques to provide detailed information of the TCR arrangement including V, D and J segments and the full sequence of CDR3 (94, 95). This high-resolution method is capable of capturing clones at extremely low frequencies. It enables to deeply profile the TCR repertoire and fully reveals the clonotype composition of T cells (96). NGS technology are able to not only monitor the TCR repertoire of a single individual on serial samples over time, but also achieve a quantitative comparison of the repertoires between two or more individuals (97). NGS improves sensitivity, reduces sequencing costs, and allows for monitoring of the immune response in large queues and at continuous timepoints.

**Figure 2:**
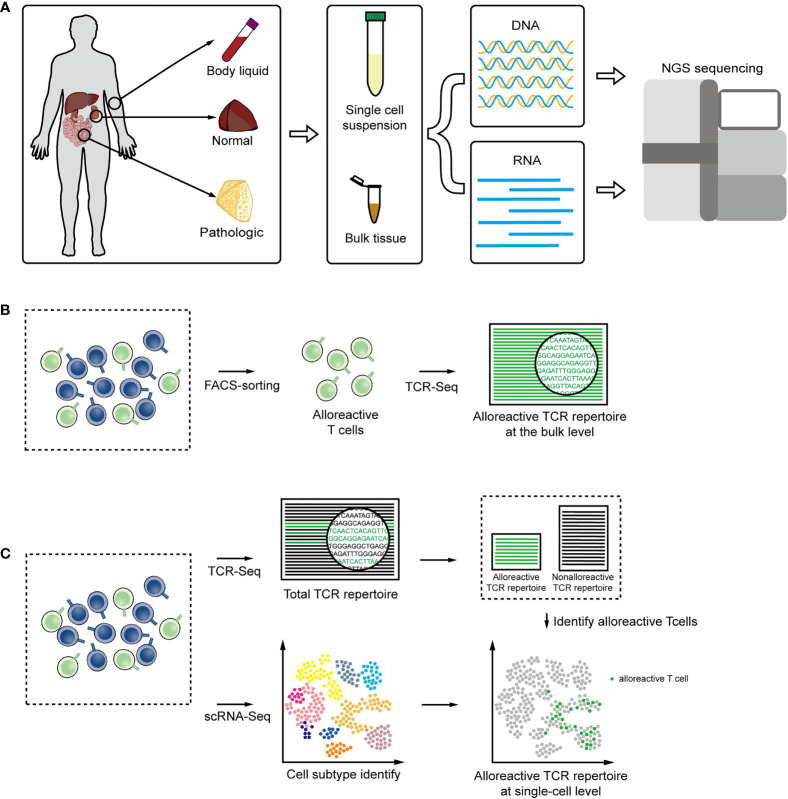
**(A)** DNA or RNA are extracted from body liquid, normal and pathologic tissues, and amplified for high-throughput sequencing technologies. **(B)** TCR sequencing combined with FACS facilitate the alloreactive TCR analysis at the bulk level. **(C)** Single-cell RNA seq combined with TCR sequencing tails the transcriptomic description and clonal dynamic of alloreactive T-cells clones.

Current high-throughput sequencing techniques that use genomic DNA (gDNA) or RNA as starting materials to construct a TCR repertoire have advantages and limitations, and the optimal choice depends on the study of interest (**Figure 2A**). Approaches based on gDNA employ multiplex amplification, a set of forward PCR primers complementary to all possible V segments and a set of reverse primers complementary to the J segments (98, 99). gDNA is stable, and there is only one copy of single gDNA in each cell to encode TCR. Therefore, gDNA-based methods allow for directly quantifying the frequencies of alloreactive clones and avoid the discrepancy in transcription levels introduced by the different activation states. In addition, initiating with gDNA skips the process of reverse transcription, that also reduces errors caused in cDNA synthesis (98). But allelic exclusion would be not considered, leading to the result that the diversity will be overestimated. And the multiplex PCR would add unavoidable amplification bias, that will interfere with the quantification of TCR sequence in the original gDNA and lead to the distortion of frequencies. RNA-based methods lean on 5′ rapid amplification of cDNA ends (RACE), which requires a fewer rounds of PCR to reduce amplification bias (98). Unique molecular identifiers (UIDs) strategy can also be applied to further reduce PCR error to achieve absolutely quantify (100, 101). Employing RNA as staring materials potentially enables to obtain the complete V and J gene sequences and provides information at nucleotide level. But RNA-based methods tend to reflect the level of gene transcription more than the absolute original cell count, and it requires high amount and quality of the starting material.

When extracting gDNA or RNA to derive informative repertoires from solid tissues or body liquids with T cells, only a small part of the sequence reads come from T cells and could correspond to TCR, so even a very high sequencing depth will cause a loss of information. Furthermore, the comparison of TCRs in different T cell subpopulations would reveal important insights into the T cell subset function, that are occluded in bulk TCR analysis. FACS sorting combined with deep TCR sequencing could enrich T cells from tissue samples or directly obtain certain T cell subgroups to facilitate more comprehensive and detailed TCR analysis (102–104). According to specific surface markers or dye, FACS sorting can also be applied to isolate alloreactive T cell subsets with cytotoxic function and particular cytokine production patterns (**Figure 2B**) (82, 105, 106).

The recent development of single-cell sequencing allows the transcriptomes of more than tens of thousands of cells to be processed simultaneously (107, 108). The combination of TCR sequencing and gene expression provides new insights into role of alloreactive T cells in alloresponse (109, 110). The total transcriptome information of a single cell can be collected and further be matched with the TCR sequence by the identical barcode sequence. In this way, each alloreactive T-cell clone can be traced through TCR and the function and development can be analyzed by gene-expression within cell (111, 112) (**Figure 2C**). Approaches based on single-cell could also obtain pairing information of the α chain and β chain to increase the ability to discover heterogeneity (102, 113, 114).

Now these approaches are widely used for characterizing the TCRs pairing and clonality and have been employed to the analysis of T-cell repertoire in various physiological or pathological states (115), including the monitoring of alloresponse by profiling total TCR repertoire dynamics and antigen-specific T cell repertoire. TCR sequencing at bulk and single-cell level allows for the more in-depth investigation of the alloreactive T cell repertoire and potential translation to clinically applicable tools.

## Immunological Characteristics of the Overall TCR Repertoire in Transplantation

Following transplantation, pre-existing alloreactive T cells are activated and clonal expanded by alloantigen from the graft. The accumulation of alloreactive T cells were observed in graft rejection (116, 117), which causes the recipient overall TCR repertoire skewed by predominant clones arising from alloresponse, and the deletion or absence of alloreactive T cells was proved in recipients within long-term graft acceptance (50, 74, 118). Therefore, information about the overall TCR repertoire would reveal the immune states of recipients after transplantation.

The changes of the diversity in the overall TCR repertoire of recipients with different clinical outcomes have been researched to reflect the T cells dynamics after solid organ transplantation (119, 120). In kidney transplantation clinical trial studies, the TCR repertoires of rejected patients were skewed related to the level of transplant lesions classified by Banff classification, while the TCR repertoires of operational tolerance patients exhibited less skewed and maintained diversity (121, 122). However, these studies only characterized the most abundant clones owing to the application of low-throughput techniques, while ignoring most of the subdominant and low-frequency ones. Methods based on the high-throughput bulk TCR sequencing provide higher sequencing coverage results to depict the entire T-cell repertoire, including TCR repertoire structure, turnover, clonality, and diversity at time points before and after transplantation.

By using the high-throughput bulk TCR sequencing method, research revealed combined kidney and bone marrow transplantation (CKBMT) recipients have a higher repertoire turnover rate than non-rejective traditional transplant recipients, which is consistent with the more effective donor-reactive T cells depletion treatment under CKBMT conditions (123). The diversity of CD4 T cells in CKBMT rejected recipients exhibited decreased tendencies after transplantation, while the diversity in tolerant recipients returned to the pre-transplantation levels. There was no difference in the diversity of CD8 T-cell repertoires was observed between groups.

In a kidney transplantation study, Alachkar et al. observed the TCR repertoire of transplant patients was relatively restricted compared with the healthy donor, which may be related to their pathology. A rapid turnover of the TCR repertoire in circulation during T cell-mediated rejection (TCMR) was reported (124), which was absent in non-TCMR patients. Additionally, the TCR repertoire of peripheral blood was highly related to that of graft during rejection suggests that noninvasive blood-derived TCR analysis has the potential to detect renal transplant rejection.

In liver transplantation (LTx), the diversity of TCR sequences was significantly decreased when acute allograft rejection occurred compared to the patients with stable allograft liver function and healthy controls, which was similar to renal transplantation (125). TCR repertoire of patients undergoing rejection appeared to have short N-additions, which may imply the recombination diversity of CDR3 during rejection. For most recipients, the diversity of TCR repertoires changed after transplantation. The overall clonality in CD8 T cells was also higher than that in CD4 T cells post-LTx, which was consistent with pre-LTx (105).

During GVHD, TCR deep sequencing analysis shows highly skewed T-cell repertoires of tissues, which matches the oligoclonal expansion pattern in previous studies about TCRs (126, 127). These T cells distribute differently from tissue to tissue in a patient, and the T cells infiltrated within solid tissues are highly individual-specific. Some studies indicated that the frequencies of clonotype from tissue and peripheral blood were not related (128, 129), but Beck et al. indicated that some clonotypes were shared (130). In fact, the TCR repertoire of peripheral blood is far more diverse and mixed than local organs, and the tissue-infiltrating T cells in circulation with low frequencies and in target tissue with high frequencies could reflect the clonal expansion in target tissues during GVHD.

In summary, the dynamics of overall TCR repertoires were related to the immune state post-transplantation, which possesses the potential to be a reference for clinical immunosuppression strategies. Additionally, the overall TCR repertoire profiling provides information about the immune status of the recipient on the waiting list before transplantation, which might be an indicator of the risk of rejection or GVHD after transplantation, but this needs further investigations. In kidney transplantation, the dominant T cell clones in the graft during TCMR could be detected in the blood and biopsy earlier, which indicates that the clonal expansion of alloreactive T cells may be much earlier than the occurrence of TCMR, and possesses the potential of being used as a biomarker for early diagnose of TCMR (124). And the entire TCR repertoires of patients with different clinic outcomes differ in terms of diversity index, physical and chemical properties, and turnover rate. These changes of TCR repertoires before or during TCMR indicate the potential of TCR repertoire to predict the rejection as a biomarker. However, the entire TCR repertoire analysis only provides information for the T-cell repertoire and leaves the antigen-specific T-cell clones profiling concealed.

## Monitoring the T Cell-Mediated Antigen-Specific Alloresponse by TCR Sequencing in Transplantation

Through tracking unique TCRs in tissues and body fluids, the expansion or reduction of antigen-specific T-cell clones can be quantified (131, 132). In the context of transplantation, tracking and monitoring graft versus host (GVH) and host versus graft (HVG) alloreactive T cells before and after transplantation in the peripheral blood, bone marrow (BM), and graft allows for a comprehensive view of the T-cell mediated alloresponse. And immunodominant clonotypes in target tissue have also aroused interest as surrogate markers for the T-cell mediated immune response in transplantation (130). Nevertheless, antigen specificity of alloreactive T-cell clones in transplantation varies from patient to patient and even from tissue to tissue, which brings great difficulty to determining the individualized alloresponse for each subject (126, 133).

MLR using donor and recipient cells could be applied to identify the alloreactive clones of each patient after transplantation. In renal transplantation, Dziubianau et al. used donor T cell-depleted peripheral blood mononuclear cell (PBMC) as stimulators in a short-term MLR with T cells in the allograft and urine post-transplantation for assessing recipient alloantigen-specific T cells in these samples (96). They reported the identification of donor-reactive clones within allografts and urine was associated with ACR. However, the frequency of these donor-reactive T-cell clones in these original samples and if it is related to ACR degree were not reported in this study.

To evaluate the deletion or accumulation of alloreactive T cells post-transplantation, an antigen-specific TCR repertoire can be constructed by carboxyfluorescein diacetate succinimidyl ester (CFSE) MLR combined with TCR-seq to quantify the frequency of alloreactive T cells. Instead of selecting alloantigen-specific T-cell clones directly in post-transplant samples as the former study, this approach obtains the alloreactive library of TCRs with pre-transplant samples MLR and monitors alloresponse prospectively. Covalently binding to amines in cells, the CFSE dye in one cell will divide in half when the cell divides into two, so the fluorescence intensity of the daughter cell is half of the original parent cell. Based on the flow cytometric analysis of MLR cultures, the cells that proliferate in response to allostimulation can be sorted.

**Figure 3:**
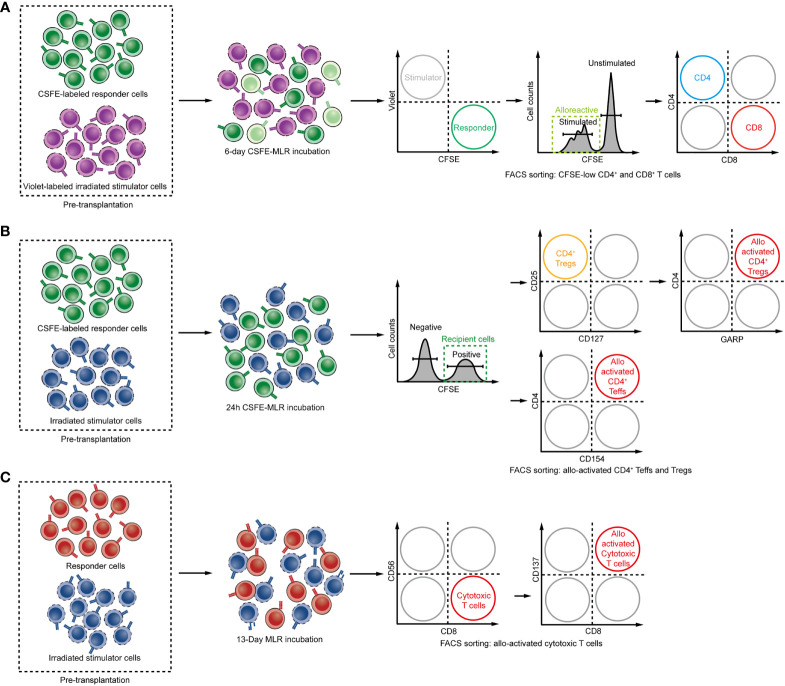
**(A–C)** Steps for determining the alloreactive TCR repertoire: Schemes for isolating alloreactive T cells in MLR. **(A)** CD137 expressing CD8 T cells are defined as alloreactive cytotoxic T cells. **(B)** The donor and recipient cells are cocultured as stimulator or responder, and the CFSE^low^ population was defined as alloreactive T cells. **(C)** Early activation marker CD154 and GARP expressing CD4 T cells and Tregs after 24 hours of MLR are defined as alloreactive CD4 Teffs and Tregs.

Bettens and colleagues obtained the alloreactive TCR repertoire through a similar one-way MLR strategy and took bystander expansion during MLR into consideration (82). They set multiple pairs of responder and stimulator cells from healthy donors with various HLA combinations to perform MLR cultures to evaluate the T cell alloresponse in human. After 13 days of MLR, the responder cells were stimulated overnight by ‘PHA blasts’, which was the autologous PBMC without irradiation and activated with PHA, and then those responder T cells exhibited activated phenotype in the second step of MLR with these autologous cells were subtracted from the allogeneic T cell pool as the bystander component. After this two-step MLR, the CD8^+^CD137^+^ subpopulation was purified and sequenced as alloreactive activated cytotoxic CD8^+^ responder T cells (**Figure 3A**). In this study, either one responder cell stimulated with two individual HLA identical healthy donor stimulator cells, or two individual responder cells from two donors stimulated with one stimulator cell, resulted in disparate alloreactive T-cell clones. This study highlighted the necessity of personalized alloreactive T-cell library construction.

Using a similar strategy, Zuber and his colleagues constructed both HVG and GVH repertoires and deleted the bystander clones by excluding 4-fold less proliferative clones in MLR compared with the original responder cell sample clone frequency. By these HVG and GVH libraries, they investigated the recipient T_RM_ replacement in graft after intestinal transplantation (ITx) and confirmed that the rate of donor T_RM_ turnover in the graft is influenced by two-way alloreactivity (117). GVH reactive clones that were enriched and persisted for long time in the graft were correlated with reduced risk of rejection. In contrast, rejection occurred when HVG reactive clones dominated in the graft and accelerated the T_RM_ turnover, and these clones acquired a kind of steady tissue-resident phenotype. Moreover, the expanded GVH reactive T cells protecting the allograft against rejection have been shown by profiling GVH clones transcriptomes using single-cell RNA sequencing combined with TCR sequencing to demonstrate a cytotoxic effector phenotype (116). They attacked recipient hematopoietic cells and make space in the BM, resulting in the long-term engraftment of donor-derived hematopoietic stem and progenitor cells (HSPCs) to the BM and finally inducing central tolerance which led to the durable chimera (134). By identifying HVG and GVH clones within the allograft, PBMC and BM, the mechanism of tolerance induction through GVH T cells was well demonstrated.

Based on the same method, Morris et al. detected fingerprints of the alloreactive T cell in CKBMT using pre-transplant materials (123). Through deep-sequencing of proliferating recipient CD4 and CD8 T cells after 6 days of MLR with donor lymphocytes as stimulators, the author obtained a mappable HVG reactive T-cell repertoire library and track these HVG clones as alloreactive T-cell clones after transplantation (**Figure 3B**). The result suggested that the dominant donor-reactive clones in *in vitro* MLR culture were most likely to expand post-transplant. By evaluating expansion or deletion of clones, reduction of circulating HVG reactive T-cell clones was observed in tolerant patients, while HVG clones were observed in nontolerant recipients, which suggested that elimination of HVG reactive T-cell clones appeared to be a mechanism of allograft acceptance in CKBMT patients. In contrast, kidney transplant recipients receiving conventional immunosuppression showed a persistent proliferation of HVG reactive T-cell clones in circulation following transplant. Similar results were observed in intestinal transplantation. The number of donor-reactive TCRs within the circulation of intestinal allograft recipients after transplantation increased (117). The successful identification of relevant donor-reactive clones following transplantation by pre-transplant MLR indicated that this method is reliable in identifying alloreactive T cells.

Unlike the expansion of circulating HVG reactive T-cell clones of conventional kidney allograft recipients, even if there is a lack of demonstrable tolerance, a decrease in circulating HVG reactive TCRs could be observed in LTx recipients (118). This may be related to the distinct distribution of T cells between liver-graft and circulation. This distinct distribution of T cell pool between liver-graft and blood was demonstrated by Mederacke et al. They used a time-saving MLR method by incubating recipient T cells sample for 24 hours with irradiated donor splenocytes as stimulators prior to transplantation, and subsequently sorted and sequenced the CD154 positive cell as alloreactive effector T cells (Teffs) (105) to construct the HVG TCR library (135, 136) (**Figure 3C**). Then they tracked HVG reactive T-cell clones at multiple time points in blood samples, and reidentified those clones in the liver biopsies. They found that TCR repertoires in circulation were distinct from that in liver-graft. But they also found the HVG clones were more correlated between allo-graft and blood in ACR patients compared with patients without ACR. They also tracked HVG Tregs, as glycoprotein A repetitions predominant (GARP) positive cell in MLR, within the peripheral blood and liver allograft in four patients with no suspected rejection, and found donor-reactive Tregs preferred to accumulate in the liver of these patients, which may be related to their function to suppress immune responses and prevent rejection (60).

In the field of transplantation, the strategies to accurately predict and recognize the rejection or tolerance are still lacking. Alloreactive T cells are considered to take responsibility for the occurrence of rejection and the failure to induce tolerance. Using an HVG or GVH TCR repertoire to identify alloreactive T cell clones provides new insights into T cell mediated allo-response and illustrates the mechanism of rejection or tolerance in transplantation, and it holds potentials for clinical usage in the future. Donor-reactive clones accumulating in the body fluid might be a biomarker for predicting rejection or GVHD in certain types of transplantation, so tracking these clones could instruct the personalized immunosuppression in clinic. The alloreactive TCR repertoire is prospectively and non-invasive in the prediction and diagnosis of rejection, and has less inter-observer bias compared to histological sectioning and staining. The combination of TCR sequencing with FACS soring or single-cell sequence can enhance posttransplant immune monitoring and immunophenotyping of alloreactive T cells in a clinical setting (**Figures 2B, C**). Additionally, some infections after solid organ transplantation could cause similar tissue damage like that in rejection and bring diagnostic challenges, so tracking the arising of donor-reactive clones referring to an alloreactive TCR repertoire can contribute to the diagnosis of posttransplant complications. Moreover, deletion of alloreactive TCRs in the blood was proved to be related to tolerance induction in certain types of transplantation, which could serve as a biomarker for the immunosuppression withdraw. The alloreactive TCR repertoire may open a new way of evaluating the establishment of immune tolerance or distinguishing tolerant patients from non-tolerant patients.

But there are challenges in translating it into clinic. The construction of alloreactive TCR repertoire is limited by the pre-transplant material and intensity of MLR. Pre-transplant MLR for alloreactive repertoire requires both donor and recipient cells before transplantation, an insufficient number of pre-transplant cells or too weak MLR may result in too few identified donor responsive clones. But it still possesses a potential of clinical application, since 10-20ml of blood can provide millions of T cells as stimulators or responders, and tens of thousands of alloreactive clones can be obtained using donor and recipient pretransplant peripheral blood samples as starting materials. For the analysis of samples with low cell number, alloreactive T cells from MLR could be expanded *in vitro* by an unbiased polyclonal expansion method to get enough cells for TCR sequencing to construct the alloreactive TCR library. And NGS is able to analyze alloreactive clones at extremely low frequencies. Additionally, several factors should be taken into consideration in alloreactive TCR library construction. Differences in allogeneic MLR coculture duration can result in different size and diversity of the alloreactive T-cell repertoire, since the alloresponse mediated by direct recognition pathway or indirect recognition pathway often occur at different periods after transplantation *in vivo*, and the overlap of TCR repertoires in these two pathways mediating the alloresponse is largely unknown in this field. And identifying alloreactive T cells by early activation markers shortens the time of MLR and can obtain enough donor-reactive T-cell clones compared with CFSE-based MLR (105, 118), but the expanded bystander T-cell clones should be removed from the alloreactive TCR library (82, 106). Also, the tracking result is limited by the post-transplant sample size, some patients under pathological conditions after transplantation, such as lymphopenia caused by GVHD or certain virus infection, may result in reduced T cell number in samples, which would cause failure in antigen-specific T cell clone tracking. Lastly, the different distribution of T-cell clones between tissues and circulation should be considered to avoid a skewed TCR repertoire in the starting T-cell repertoire, to better serve the research purpose by targeting as much as possible antigen-specific T-cell clones in constructed alloantigen-specific TCR repertoire.

## The Potential of Using Computational Tools to Characterize the TCR Repertoire

Although the biochemical and molecular basis of TCR recognition of allogeneic peptide/MHC is becoming clearer, the ignorance about specificities of alloreactive T cells still limits the biological insights into alloreasponse. Existing computational tools for alloreactive TCR repertoire annotation allow both matching against databases of known antigen specificities and clustering of TCR sequences through algorithms (109). With these public tools, annotating TCR repertoire data in alloresponse and integrating alloantigen-specific clones can be more efficient.

The international Immunogenetics Information System (IMGT) includes a database that providing reference sequences for individual gene of T-cell receptors, and annotations of known gene segments (137). It allows user to submit nucleotide sequences to identify the V, D and J genes and alleles and characterize clonotypes. To share data and annotate TCR repertoires with predicted antigen specificities, a series of databases across studies were established (138, 139) (**Table 1**). In McPAS-TCR, TCR sequences associated with various antigens were manually arranged according to published literature (139). And VDJdb collects published T-cell specificity assays and lists TCR sequences with experimentally verified epitope specificities as well as their MHC restrictions (138). Some databases such as VDJServer and iReceptor, places extra emphasis on sharing data on adaptive immune receptor repertoire (AIRR), and provides online AIRR-seq data of interest for conjoint analysis and data mining (78, 142). The AIRR Community was formed to solve problems in AIRR sequencing studies (143). To meet the need for queries across repositories, standard procedures for AIRR-seq data acquisition, storage, submission, annotation, and sharing are established and a common file format is proposed, which will create a unified environment of data analysis for individual researchers.

**Table 1:** Summary of TCR databases.

	Function	Species	Available information	Source
**IMGT (** 140 **)**	A system that bridge biological and computational spheres in bioinformatics	Human, rat, goat, monkey, dog, rabbit, pig, cat, sheep and frog	Locus, genes, alleles, proteins, probes, structures, clinical entities	http://www.imgt.org/IMGTrepertoire/
**McPAS-TCR (** 139 **)**	A manually curated catalogue of pathology-associated TCRs	Humans and mice	Epitope, disease condition, T cell type, tissue, source organism, MHC restriction, assay type	http://friedmanlab.weizmann.ac.il/McPAS-TCR/
**VDJdb (** 138, 141 **)**	A database of TCRs with known antigen specificity	Humans, mice and monkeys	Epitope, antigen, MHC, HLA type, assay types and sequencing methods	https://vdjdb.cdr3.net/
**VDJserver (** 142 **)**	A Cloud-Based Analysis Portal and Data Commons for Immune Repertoire Sequences and Rearrangements	Human and mice	Gene usage, diversity, length, amino acid utilization, and physicochemical properties of CDR3 patterns	https://vdjserver.org/
**iReceptor (** 78 **)**	A platform for querying, analyzing and downloading antibody/B-cell and T-cell receptor repertoire from multiple independent repositories	Human and mus musculus	Study types, organisms, diagnoses, tissues, PCR targets and target substrates	http://ireceptor.irmacs.sfu.ca/

The TCRs of T cells may share antigen specificity and recognize the same peptide-MHC ligands even if their amino acid or nucleotide sequence are different (144). TCR clustering tools cluster TCRs of similar specificity by algorithms related to the combination of different factor (**Table 2**). Dash et al. developed TCRdist based on the structural information on pMHC binding (145). This analytical tool can quantify the similarity of TCRs according to distance on the space and assign unknown TCRs to epitope-specific receptors repertoires. A distance score is computed by comparing all CDRs using a similarity-weighted Hamming distance. Although TCRdist is considered to be an effective high-clustering metric, the requirement for pairwise Smith–Waterman (SW) alignment on both the CDR3 sequences and the variable gene alleles limits the size of the TCR repertoire that can be clustered. GLIPH (grouping of lymphocyte interactions by paratope hotspots) can retrieve and group similar TCRs of common specificity within an individual or across a group of donors through scoring based on multiple factors, including the motif, CDR3 length, shared HLA among contributors (148). This clustering algorithm can be used for the detection of antigen-specific TCRs and predicting the specificity of a new TCR, with no need of knowing the epitope. Those algorithms classify TCRs by calculating similarity, which is inefficient and strenuous to deal with large cohorts of TCR-seq samples. As an improved version, GLIPH2 greatly accelerates the analysis of TCR sequences with high clustering efficiency and accuracy (146). Apart from a series of scores for clustering, the fisher-exact test is introduced to filter given motifs for significance. In GLIPH2, a TCR sequence can be assigned to more than one cluster. iSMART allows comparison between CDR3s of different length and imposes a gap penalty. It also has high average purity for large clusters and fast speed for calculation. CDR3s will be first ordered the by length, and then compared in pairs (25). Geometric Isometrybased TCR AligNment Algorithm (GIANA) focuses on fast handling large-scale TCR datasets and increases efficiency while maintaining the same level of accuracy (147). These TCR clustering tools can divide large numbers of TCR sequences into groups with shared specificity and identify antigen-specific TCR groups according to the known TCR sequences in the groups (149).

**Table 2:** Summary of methods for linking TCR-antigen specificity.

Method	Function	Approach	Source
**TCRdist (** 145 **)**	Matching TCR repertoire against a database of TCR sequences	Sequence similarity distance	https://tcrdist3.readthedocs.io/en/latest/index.html
**GLIPH2 (** 146 **)**	Clustering TCRs that are predicted to bind the same pMHC	K-mer enrichment-based detection of TCR motifs	http://50.255.35.37:8080/
**iSMART (** 25 **)**	Grouping similar TCRs into antigen-specific clusters	Pairwise local alignment on T cell receptor CDR3 sequences	https://github.com/s175573/DeepCAT/blob/master/iSMARTm.py
**GIANA (** 147 **)**	TCR clustering and multi-disease repertoire classification	Nearest neighbor search in the high-dimensional Euclidean space	https://github.com/s175573/GIANA

A large set of TCR-antigen specificity data is required for understanding the structure and features of alloresponse due to the huge diversity of TCR sequences and pMHC. But the cells driving alloresponse may be small in number, and the alloreactive TCR repertoires in each small cohort may be drowned out in sequencing reads. Integrated online databases facilitate the accumulation and complex analysis of alloreactive TCR repertoire. Alloreactive TCR records can be submitted and arranged across species and tissue from multiple assays, including the CDR3 sequence, antigen, epitope, MHC and clinical presentation data, to help the identification and characterization of alloreactive TCRs and the epitopes they recognize. Algorithms help to further investigate specificities of alloreactive TCRs by calculating similarity. A deeper understanding of the alloreactive TCR repertoire will aid in the development of future immunomodulatory therapies in solid organ transplantation. And future studies in identifying allogeneic pMHC epitopes using these tools will enable the characterization of alloreactive TCR repertoires, which will provide further insights into the fundamental basis of alloresponse in biophysics and structure.

## Conclusions

The development of NGS technology has brought improvements in TCR high-throughput and applications by making TCR repertoire analysis a basic tool for T-cell study in healthy physiological conditions and various pathological conditions. TCR sequencing of thousands to millions of cells shows the complexity and diversity not only of the whole TCR repertorie but also specific subset of T-cell clones in body liquids and various tissues. Linking the TCR repertoire with gene expression profiles led to further information, enabling to trace of specific T cells developmental fate and biofunction. The development of web databases and computational methods greatly expanded the available TCR information.

In the setting of transplantation, the dynamics of the overall TCR repertoires after transplantation are closely related to the immune status after transplantation. The diversity of repertoires and the rate of turnover is related to rejection or tolerance. The combination of MLR and TCR repertoire to track donor or recipient antigen specific T cells has been demonstrated as reproducible and sensitive. This method opens up a new path to monitor the T cell-mediated alloresponse after transplantation. Using the responders and stimulators in one-way MLR from the donor and recipient cells materials before transplantation, a fingerprint of the HVG or GVH reactive T-cell clones during the alloresponse can be defined and tracked after transplantation. By tracking these clones, the clonal deletion or expansion of HVG and GVH reactive T cells reveals antigen-specific T-cell clonotypes are closely related to the immune status of the patients after transplantation and have a correlation with transplant outcome. Under certain circumstances, the clinical translation of TCR repertories analysis in the peripheral blood would provide valuable information for treatment decisions. Hence, TCR repertoire analysis can noninvasively assist the diagnosis and treatment of transplant patients. In general, longitudinal monitoring of changes in the alloreactive clone size and overall TCR repertoire after transplantation can be used as a biomarker to predict tolerance or rejection in certain types of transplantation, eventually allowing individualization of immunosuppression in the clinic. And the characterization of alloreactive TCR repertoires and epitopes in the future will provide mechanistic insights into alloresponse.

## Author Contributions

GT wrote the manuscript and designed the figures. GL and ML edited and revised the manuscript. All authors contributed to manuscript revision, read, and approved the submitted version.

## Funding

Grants from Natural Science Foundation of China (grant number: 81901627 and U20A20360) will provide financial support for the open access publication fees of this paper.

## Conflict of Interest

The authors declare that the research was conducted in the absence of any commercial or financial relationships that could be construed as a potential conflict of interest.

## Publisher’s Note

All claims expressed in this article are solely those of the authors and do not necessarily represent those of their affiliated organizations, or those of the publisher, the editors and the reviewers. Any product that may be evaluated in this article, or claim that may be made by its manufacturer, is not guaranteed or endorsed by the publisher.
